# Acute Hemorrhagic Myelitis in an Adolescent With COVID-19: A Case Report and Review of Literature

**DOI:** 10.7759/cureus.20553

**Published:** 2021-12-20

**Authors:** Yazid Maghrabi, Saleh S Baeesa

**Affiliations:** 1 Neurosciences, King Faisal Specialist Hospital and Research Centre, Jeddah, SAU

**Keywords:** sars-cov2, fever, children, hemorrhagic, myelitis, covid-19

## Abstract

Coronavirus disease 2019 (COVID-19) infection is considered a multisystem disease rather than solely affecting the respiratory system. In addition, many reports have described neurological manifestations of this disease; yet reports on spinal cord involvement, especially in pediatrics, are still limited. We describe a case of a 15-year-old male with COVID-19, who presented with sudden paraplegia and urinary incontinence, preceded by a two-day history of fever. Upon clinical and radiological assessment, he was diagnosed with acute hemorrhagic myelitis. A remarkable motor improvement upon a nine-month follow-up was perceived. Our case illustrates that serious complications can arise even though COVID-19 causes milder disease in pediatrics. We advocate for vaccinating the pediatric population to prevent such occurrences.

## Introduction

A new coronavirus was identified, severe acute respiratory syndrome coronavirus-2 (SARS-CoV-2), which results in a respiratory illness called coronavirus disease 2019 (COVID-19) [1]. Since the first reported cases in December 2019, the number of cases had grown exponentially, making COVID-19 a global pandemic with significant mortality and morbidities. Most infected patients have a variable spectrum of clinical presentations varying from mild symptoms with fever dry cough to severe respiratory disease [2]. However, a recent meta-analysis has shown that the pediatric population experiences milder disease with atypical clinical features [3].

There have been growing numbers of published case reports and case series detailing various neurological manifestations of COVID-19, reflecting the scientific community's growing interest in understanding this novel disease and its effect on the nervous system. It has been mentioned that COVID-19 spinal complications have been reported widely in the literature, as claimed by Mondal and colleagues [4]. On the other hand, Schulte et al., in their systematic review of acute myelitis in individuals with COVID-19, have argued that association does not necessarily mean causation and that most of the complications occur either during or after the infection [5]. Reports of spinal complications in pediatric patients with COVID-19 are scarce.

We herein present a case of a 15-year-old male presented with acute hemorrhagic myelitis associated with COVID-19, with a review of the current published literature.

## Case presentation

A 15-year-old male with no known trauma or comorbidities presented to the emergency department with a rapidly progressive lower limbs weakness and urinary and bowel incontinence for two days. His parent reported a five-day history of fever and cough that preceded his presentation. One day earlier, he had an oropharyngeal swab for severe acute respiratory syndrome coronavirus 2 (SARS-CoV-2), and the polymerase chain reaction (PCR) was positive conﬁrming COVID-19. 

On physical examination, his heart rate was 56 beats/min with a regular rhythm, blood pressure of 100/65 mmHg, normal respiration of 24/min with oxygen saturation (O2-Sat) of 95% on room air, and mild pyrexia (37.6 degrees C); general examination of cardiorespiratory and abdominal systems was unremarkable. He was conscious, alert, oriented, with intact cranial nerves. His upper extremities power was 3/5, according to medical research council (MRC) score, with decreased tone and reflexes. The lower extremities were flaccid with motor strength of 0/5, absent reflexes, and equivocal Babinski sign. Urinary bladder catheterization was performed, which drained 800 mL, and examination for bulbocavernosus reflex was positive.

**Figure 1:**
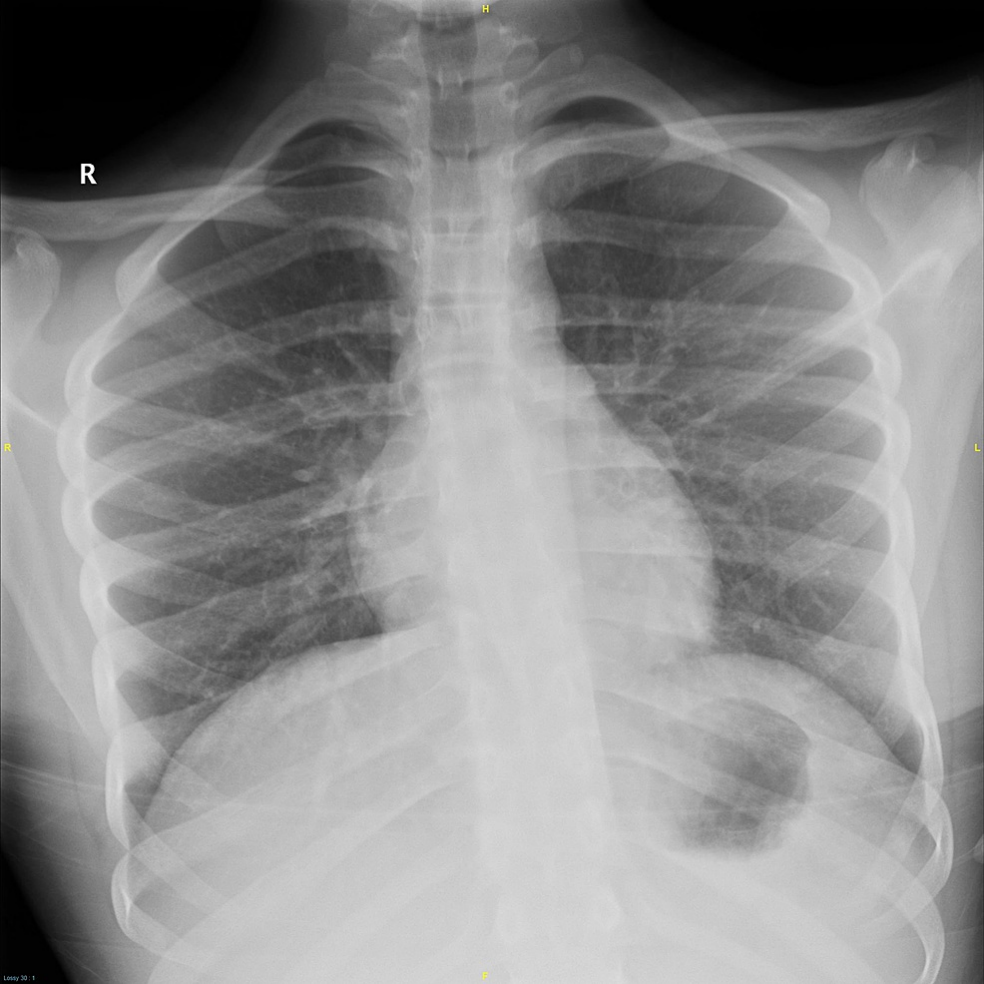
Chest x-ray on admission was normal.

Emergency brain and cervical spine CT scan studies were unremarkable, and his chest x-ray was normal on admission (Figure 1).

Initial laboratory tests revealed a normal white blood cell count, normal hemoglobin, and normal platelet count (Table 1). The coagulation profile revealed normal partial thromboplastin time (PTT), normal prothrombin time (PT), and normal international normalized ratio (INR) (Table 1). In addition, inflammatory markers revealed elevated C-reactive protein (CRP) and high lactate dehydrogenase (LDH) (Table 1). The autoimmune panel for lupus and the antiphospholipid syndrome was negative.

**Table 1:** Patient’s initial laboratory values with corresponding reference range CBC: Complete Blood count, WBC: White Blood Cells, PTT: Partial thromboplastin time, PT: prothrombin time, INR: international normalized ratio, CRP: C-Reactive Protein, LDH: Lactate Dehydrogenase, CSF: Cerebrospinal Fluid

Laboratory test	Value	Significance	Reference Range
CBC			
WBC Count	5.42 x 10^9^/L	Normal	4.5-11 x 10^9^/L
Hemoglobin	14.7 g/dL	Normal	13.5-17 g/dL
Platelets Count	197 x 10^9^/L	Normal	150-400 x 10^9^/L
Coagulation Profile			
PTT	33.8 sec	Normal	33.9-46.1 sec
PT	14.1 sec	Normal	12.7-16.1 sec
INR	1.06	Normal	0.97-1.30
Inflammatory Markers			
CRP	12.79 mg/dL	High	<0.3 mg/dL
LDH	277 U/L	High	100-250 U/L
CSF Analysis			
Cell Count	3 cells/mm^3^ (Lymphocytes)	Normal	0-8 cells/mm^3^
Protein	623.6 mg/dL	High	15-16 mg/dL
Glucose	51 mg/dL	Normal	50-80 mg/dL

**Figure 2:**
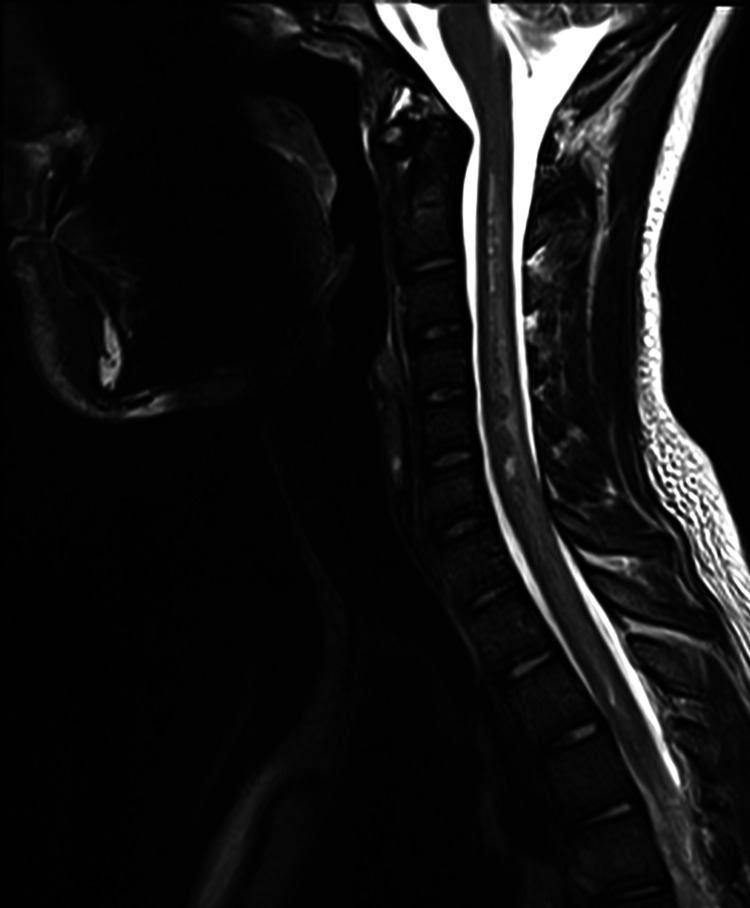
Sagittal T2-weighted MRI of the cervical spine demonstrating swelling and edema of the spinal cord.

**Figure 3:**
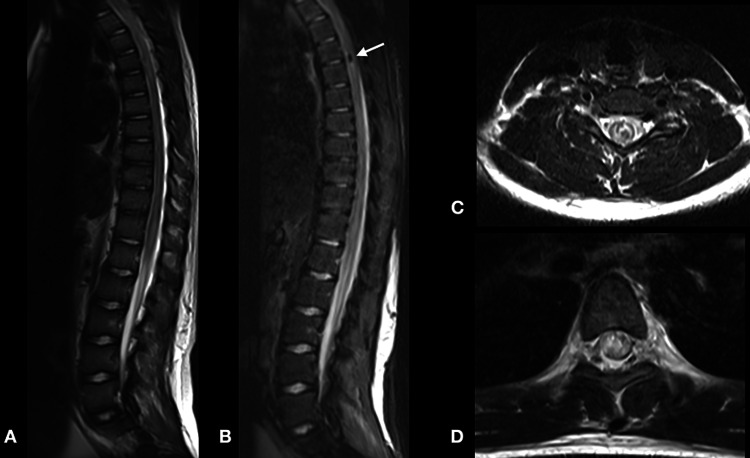
Sagittal T2-weighted MRI scans of the thoracic spine (A, B) and axial T2-weighted MRI scans (C, D) revealed the extensive spinal cord edema and signal changes suggestive of subacute hemorrhagic changes (white arrow).

Urgent brain and whole spine MRI revealed no intracranial abnormality; however, there was extensive hypointense signal involving the cervical spine and the entire length of the spinal cord in the T2-weighted image representing edema, with some areas of hypointense spots representing blood products (Figures 2, 3A-3D). On the T1-weighted image, there were multiple intramedullary hyperintense lesions of the spinal cord from the level of the second cervical segment to the conus medullaris representing early subacute hemorrhage (Figures 2, 3A-3D).

**Figure 4:**
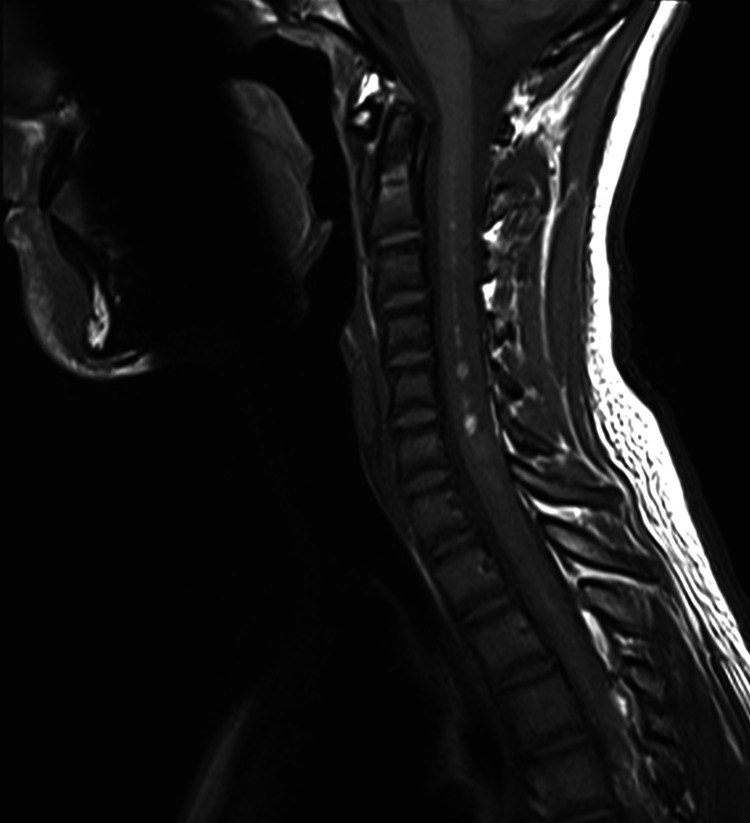
Sagittal T1-weighted MRI of the cervical spine demonstrating a bright intramedullary signal of subacute hemorrhage.

**Figure 5:**
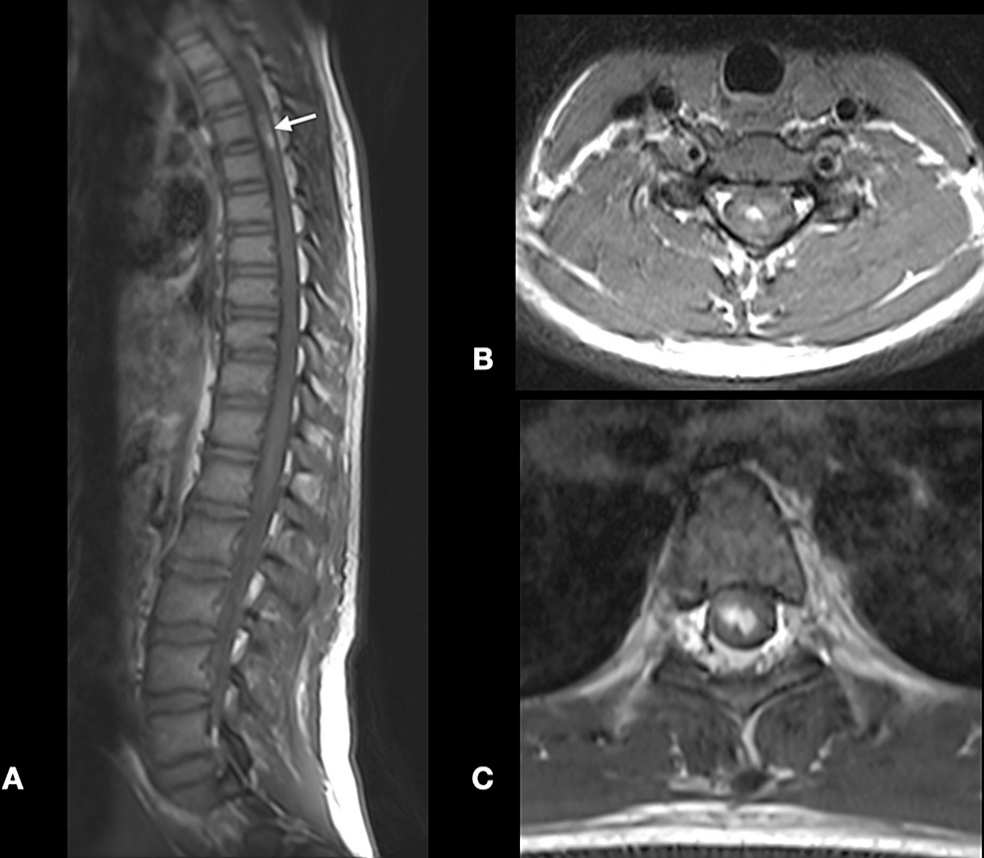
Sagittal T1-weighted MRI scans of the thoracic spine (A) and axial T2-weighted MRI scans (B, C) revealed the signal changes suggestive of subacute hemorrhagic changes (white arrow).

On the T1-weighted image, there were multiple intramedullary hyperintense lesions at the cervical and thoracic segments representing early subacute hemorrhage. In addition, there was abnormal enhancement following administration of intravenous contrast (Figures 4, 5A-5C). 

A lumbar puncture was done for cerebrospinal fluid (CSF) analysis that showed clear colorless fluid under normal opening pressure. The cell count was normal, differential showed predominantly lymphocytes, increased protein, and normal glucose (Table 1). CSF culture was negative for bacterial and fungal organisms. CSF was sent for PCR which was negative for SARS-CoV-2. In addition, mycoplasma, toxoplasma, parvovirus, cytomegalovirus, Epstein-Barr, Varicella zoster, Herpes simplex, hepatitis, and HIV were ruled out.

The patient was admitted to the pediatric ICU for cardiorespiratory and neurological monitoring. He was started on dexamethasone 4 mg every six hours and started on intravenous immunoglobulin therapy (IVIG) of 0.4g/kg for three days, followed by plasma exchange with no remarkable neurological improvement. 

**Figure 6:**
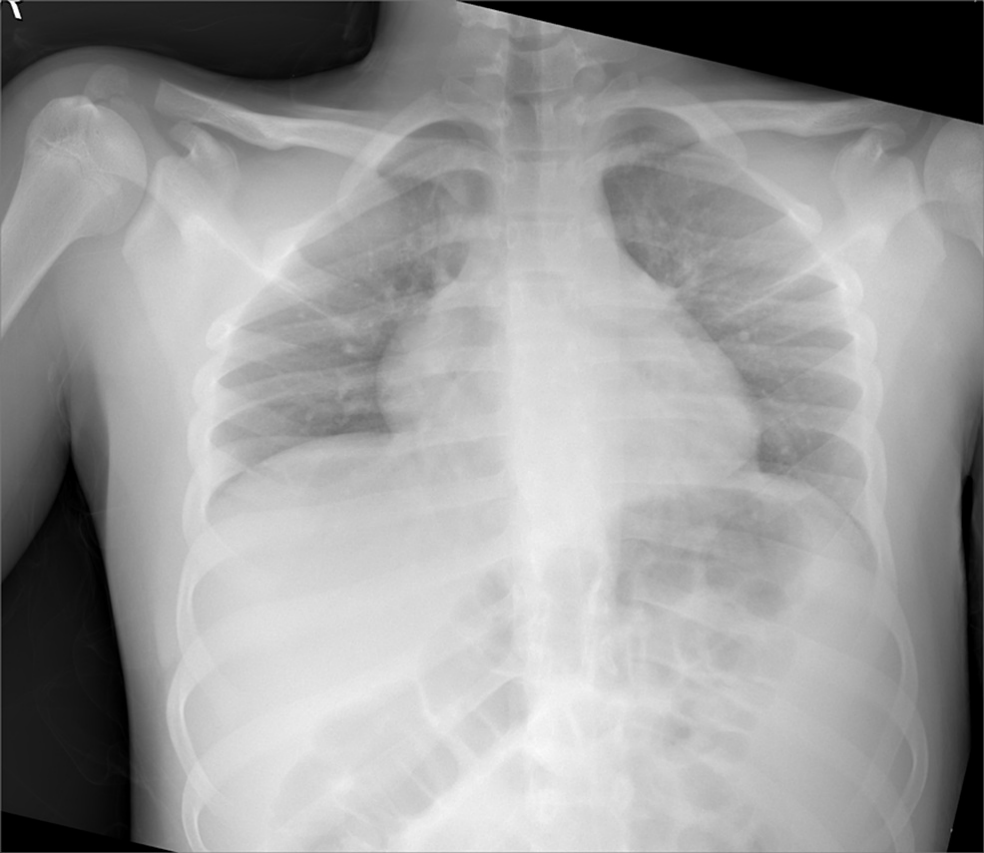
Chest x-rays revealed severe SARS-CoV-2-induced ARDS. ARDS - adult respiratory distress syndrome

After five days, the patient had severe dyspnea with hypoxemia due to SARS-CoV-2-induced adult respiratory distress syndrome (ARDS-SARS-CoV-2). His chest x-ray revealed significant consolidation (Figure 6).

He was managed with non-invasive ventilation (NIV) in a prone position with a high-flow nasal cannula (HFNC) and intensive chest physiotherapy with considerable improvement in oxygenation. In addition, he was started on ceftriaxone (1 gm intravenously every 12 hours) and vancomycin (800 mg intravenously for 12 hours) for 15 days. The patient was discharged after 21 days to a rehabilitation facility with good general and respiratory conditions; however, there were remarkable changes in his neurological function. 

**Figure 7:**
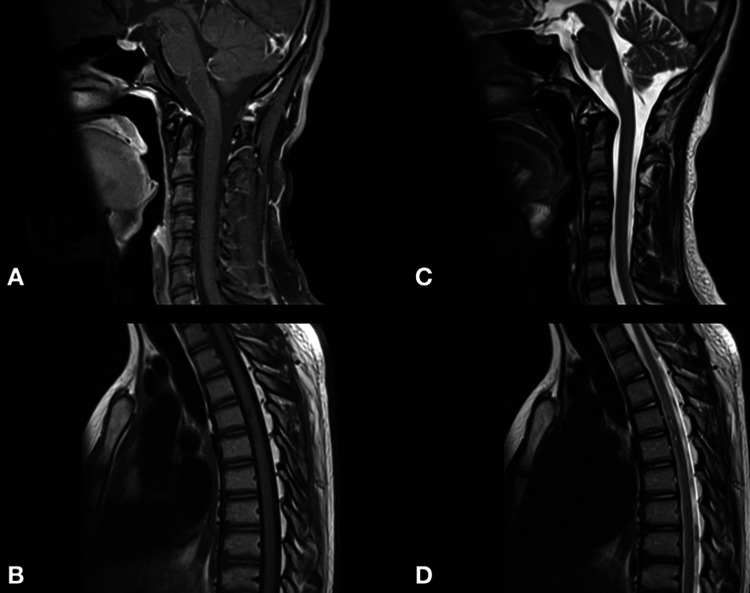
Follow-up T1-weighted (A, B) and T2-weighted (C, D) MRI scan of the cervical and thoracic spine revealed resolution of the hemorrhagic changes.

At a nine-month follow-up, he presented to the outpatient clinic with remarkably improved ambulation with mild assistance and regained sphincteric function. In addition, a follow-up MRI revealed a remarkable resolution of spinal cord edema (Figures 7A-7D).

## Discussion

COVID-19 pandemic has harshly affected the world population, leading to extensive research and studies by clinicians and researchers worldwide to understand better the pathophysiology of how such a virus can cause disastrous complications [5]. It has been observed that immunological processes affect multiple organ systems following COVID-19 [5]. It is well known that the immunological processes play a significant role in developing neurological manifestations following the infection, where it leads to several manifestations affecting the central nervous system and peripheral nervous systems [6]. Reports of spinal involvement in patients with COVID-19 are scarce, especially in pediatric and adolescent populations.

Mondal and colleagues have conducted a systematic review to quantify reports of spinal involvement in COVID-19 patients [4]. Their search yielded 21 cases of different types of myelitis, only one of which is of a pediatric patient [5]. Moreover, there was no mention of cases of acute hemorrhagic myelitis. Another prospective study conducted in Saudi Arabia has found that out of 498 patients with COVID-19, none had presented with myelitis [7]. This indicates that spinal involvement in patients withCOVID-19 is rare and less so among pediatric patients.

Multiple reports have suggested the pathological mechanisms in developing spinal complications following COVID-19: direct neurotropism or immune-mediated injury [4]. Regarding the latter, Schett and associates have mentioned that due to cytokine storm induced by the infection, there might be activation of glial cells that might lead to neurological manifestations [8]. Furthermore, it has been postulated that the cytokine storm can also lead to disruption of procoagulant balance resulting in microthrombi and disseminated intravascular coagulopathy, which could result in life-threatening hemorrhage [9]. The latter could explain the imaging finding in our patient, in which he had subacute blood in the spine.

As per reported cases in the literature, the clinical presentation of spinal involvement in COVID-19 patients ranged from motor weakness, sensory affection, and some cases presented with autonomic dysfunction manifested by sphincter problems [4]. The only pediatric case included in the review of Mondal and colleagues was a case reported by Kaur et al. of a three-year-old girl who presented with flaccid quadriparesis followed by quadriplegia, which led to respiratory failure and the requirement of airway support [4,10]. Another case of an 11-year-old female was reported in the literature, who presented with severe flaccid paralysis that required prolonged intensive care and mechanical ventilation [11]. We can infer from these cases that even though the COVID-19-affected pediatric population does not present with severe respiratory symptoms and signs, neurological complications can be devastating. Our present case is no exception, as the patient only had a mild course of respiratory symptoms followed by progressive neurological deterioration in the form of lower limbs weakness and loss of sphincter control.

In regards to the laboratory findings, these cases should be investigated as any case of myelitis by doing routine lab tests, including complete blood count (CBC), renal profile, coagulation profile, and inflammatory markers. Autoimmune screening and other viral screening, along with COVID-19 nasopharyngeal swabs have to be performed. Furthermore, a CSF sample should be taken for analysis. Our patient had increased protein in his CSF sample only, in contrast to other published reports, where they showed elevated cell count along with protein [3]. Magnetic resonance imaging is the modality of choice to visualize spinal lesions, especially in this population, emphasizing T2-weighted fast spin-echo and short-tau inversion recovery (STIR) sequences [12].

Back in the peak of the first COVID-19 wave, there was no clinically effective therapy for COVID-19 complications. Most patients with spinal involvement received different types of corticosteroids such as intravenous methylprednisolone, intravenous dexamethasone, and oral prednisolone [3]. Other patients underwent IVIG, plasma exchange, or off-label use of different antiviral drugs [3]. Our patient was started on multiple doses of IV dexamethasone and IVIG without any improvement in neurological function.

Our case is considered unique as it is the first reported case of spinal involvement in an adolescent presenting with acute hemorrhagic myelitis. To our knowledge, no similar cases have been reported in the literature.

## Conclusions

Pediatric and adolescent populations do not exhibit severe respiratory COVID-19 related symptoms with related complications being rare. However, COVID-19-related neurological complications, especially spinal involvement, are serious. Thus, prevention is the best treatment with vaccinating the pediatric population in order to prevent such occurrences.
